# Elevated Levels of IFN-γ in CSF and Serum of Patients with Amyotrophic Lateral Sclerosis

**DOI:** 10.1371/journal.pone.0136937

**Published:** 2015-09-02

**Authors:** Juan Liu, Lina Gao, Dawei Zang

**Affiliations:** 1 Microbiology Group, Department of Laboratory Medicine, Tianjin First Center Hospital, Tianjin Medical University, Tianjin, China; 2 Department of Neurology, Tianjin First Center Hospital, Tianjin Medical University, Tianjin, China; University of Florida, UNITED STATES

## Abstract

**Objectives:**

To explore whether the levels of IFN-γ in cerebral spinal fluid (CSF) and serum are elevated in ALS patients and to analyze the correlations between the IFN-γ levels and disease progression.

**Methods:**

CSF and serum samples were obtained from 52 ALS patients and 31 non-ALS patients. The levels of IFN-γ in CSF and serum were assessed, and disease progression parameters, including the disease interval (months from onset, MFO), the revised ALS Functional Rating Scale (ALSFRS-r) score and the disease progression rate (DPR) were analyzed by registered neurologists. All samples were measured using a commercial enzyme-linked immunosorbent assay. Statistical analyses were performed using Prism software.

**Results:**

Compared to the non-ALS patients, the ALS patients displayed significantly increased levels of IFN-γ in both CSF and serum, and these values consistently correlated with disease progression.

**Conclusions:**

These results demonstrated that IFN-γ in CSF may serve as a biomarker of ALS differentiation and progression. CSF IFN-γ was a more reliable biomarker of disease diagnosis and progression than serum IFN-γ.

## Introduction

Amyotrophic lateral sclerosis (ALS) is a progressive neurodegenerative disease, but its mechanisms of pathogenesis remain incompletely understood. Studies of ALS patients have found that the levels of certain cytokines and growth factors in cerebral spinal fluid (CSF) and serum of ALS patients markedly change with the progression of this disease [1–3], this evidence suggested that cytokine may play a critical role in the pathogenesis of ALS. Thus, studies of cytokines, especially in CSF [4] may represent a tool for ALS diagnosis and evaluating disease progression.

IFN-γ is a potent pro-inflammatory cytokine proposed to contribute to motor neuron death [5], Treatment with a neutralizing anti-IFN-γ antibody can delay motor neuron decline [6]. Kwon *et al* co-cultured the peripheral blood mononuclear cells (PBMCs) with human mesenchymal stem cells isolated from healthy controls and ALS patients, and they found that the mRNA level of IFN-γ was significantly increased in ALS patients compared with controls [7]. Takahisa *et al* found an increased level of IFN-γ in CSF of ALS patients [8]. These studies suggested that CSF IFN-γ could play an important role in ALS; however, a study from Australia reported that IFN-γ was not detectable in ALS patients [9]. The variability of these findings may be due to differences on methodology or the geographical distribution of the examined patients. To date, there is little knowledge concerning how IFN-γ is associated with ALS progression. In particular, the profile of this protein in CSF and serum of Chinese ALS patients with either bulbar or limb onset remains to be elucidated. Thus, the aims of present study are to determine the levels of IFN-γ in Chinese ALS patients, to analyze the association of the IFN-γ levels with disease progression and the site of onset, and to evaluate whether IFN-γ may serve as an indicator of disease diagnosis and differentiation.

## Materials and Methods

### Patients

This study was approved by The Clinical Experimentation Committee of Human of Tianjin First Center Hospital (YZXH10316). 52 ALS patients (ALS) and 31 non-ALS patients (non-ALS) were included in this study after providing written informed consent from January 2010 to April 2014 in the inpatient neurological wards of Tianjin First Center Hospital. All ALS patients met the revised EI Escorial criteria for clinically definite or probable ALS, 14 patients experienced bulbar onset and 38 patients experienced limb onset. The mean age and gender distribution do not differ significantly between ALS patients and non-ALS patients (P>0.05). Non-ALS patients exhibited tension-type-headache (n = 18), hypokalemic paralysis (n = 3), low intracranial pressure (n = 7), or CSF leakage (n = 3). Between 8AM and 12 noon, peripheral whole blood and CSF samples were obtained via venipuncture and lumbar puncture respectively, at the time of CSF examination as part of a diagnostic evaluation. The diagnosis of ALS and the evaluation of the revised ALS functional rating scale (ALSFRS-r) score and the disease progression rate (DPR) were performed for each ALS patient by registered neurologists before the lumbar puncture. The months from onset (MFO) which means “interval from the observation of initial symptoms to diagnosis (months)” was used to calculate the DPR as follows: DPR = (48−ALSFRS-r score at time of diagnosis)/interval from the observation of initial symptoms to diagnosis (months). We followed up the surviving ALS patients via phone calls or clinic visits. Detailed informations regarding the ALS and non-ALS patients are summarized in Table 1.

**Table 1:** Summary of samples from ALS patients (ALS) and from non-ALS patients (Non-ALS).

Sample information	ALS	Non-ALS
**Cases (M/F)**	52(36/16)	31(19/12)
**Clinically definite/probable**	35/17	N/A
**Clinical onset, B/L**	14/38	N/A
**Age (years, mean±se)**	52.01±1.81	49.42±2.93
**MFO (months)**	19.17±1.95	N/A
**DPR**	1.41±0.18	N/A
**ALSFRS-r score**	36.23±0.81	N/A
**IFN-γ (pg/ml) (CSF)**	348.83±15.32* #	172.22±17.58
**IFN-γ (pg/ml) (Serum)**	280.66±15.44*	136.42±14.79

M: male; F: Female. B: bulbar onset; L: limb onset; MFO: interval from the observation of initial symptoms to diagnosis; DPR: disease progression rate; ALSFRS-r: Amyotrophic Lateral Sclerosis Functional Rating Scale revised.

* indicates a significant difference (p<0.01) in the IFN-γ levels between ALS and Non-ALS.

# indicates a significant difference (p<0.01) in the CSF IFN-γ levels compared with the serum IFN-γ levels; mean±se.

### Serum and CSF samples collection

Blood samples were drawn from all patients (52 ALS patients and 31 non-ALS patients) and centrifuged at 3000 rpm at 4°C for 10 minutes within 2 hours. The supernatants were stored at -80°C until the beginning of the experiment. The average time from blood withdrawal to centrifugation was 52.68±7.27 minutes. CSF samples were simultaneously obtained from 52 ALS and 31 non-ALS patients and immediately centrifuged at 800 rpm at 4°C for 5 minutes. The supernatants were stored at -80°C until the beginning of the experiment; the average time from lumbar puncture to centrifugation was 21.13±5.46 minutes.

### ELISA for IFN-γ

The serum and CSF concentrations of IFN-γ were measured via enzyme-linked immunosorbent assay (ELISA) using a commercial ELISA kit (Human IFN-γ Immunoassay Kit; R&D Systems, USA). In accordance with the manufacturer’s instructions, a standard series of 0 pg/ml, 75 pg/ml, 150 pg/ml, 300 pg/ml, 600 pg/ml and 1200 pg/ml were used. After preparing reagents, samples and standards, IFN-γ was added to Enzyme wells that were pre-coated with the anti-IFN-γ monoclonal antibody; 100 μl/well of a standard and 50 μl/well of Streptavidin-HRP were added to the Standard wells, whereas 100 μl/well of the sample was added to the test well, followed by the addition of 50 μl/well of IFN-γ labeled with biotin and 50 μl/well of Streptavidin-HRP and incubation for 60 min at 37°C. Then, the plates were washed five times with the wash solution to remove the unbound enzyme and incubated in 50 μl/well of chromogen solution A and 50 μl/well of chromogen solution B for 10 min at 37°C shielded from light. After incubation, 50 μl/well of stop solution, which terminated the reaction, was applied to visualize the reaction products. The optical density (OD) was measured at a wavelength of 450 nm using a BioTek Synergy 2 microplate reader (BioTek, USA) within 10 min. According to the standard concentrations and the corresponding OD values, a linear regression was calculated for the standard curve, and then the sample concentrations were obtained based on the OD values of the sample according to the regression equation.

### Statistical Analysis

All statistical analyses and graphs were performed using GraphPad Prism (Version 4.0, GraphPad Software Inc., San Diego, USA). Normal distributions of datasets were checked by quitie-quitile (Q-Q) plots. The results were analysed after testing for normal distribution. Unpaired, independent 2-tailed Student t-tests were used to examine the differences between groups. Pearson’s correlation was used for statistical correlation analysis. The statistical data were expressed as the mean±se, statistical significance was defined as a p-values below 0.05.

## Results

In total, 52 ALS patients and 31 non-ALS patients were involved in this study. The ALS and non-ALS patients did not differ in age and gender distribution (P>0.05). The clinical diagnosis, clinical onset site, MFO, DPR and ALSFRS-r of the ALS patients are shown in Table 1. The levels IFN-γ in CSF (348.83±15.32 pg/ml and 172.22±17.58 pg/ml, respectively) and serum (280.66±15.44 pg/ml and 136.42±14.79 pg/ml, respectively) were significantly different between the ALS patients and the non-ALS patients (P<0.01), and a difference was detected between the CSF (348.83±15.32 pg/ml) and serum (280.66±15.44 pg/ml) IFN-γ levels in the ALS group (P<0.01, Table 1).

The 52 ALS patients were further divided into two subgroups according to the MFO: less than 12 months (ALS<12 m) or equal to or more than 12 months (ALS≥12 m). There was no difference in age and gender distribution between the two ALS subgroups and the non-ALS patients (P>0.05, Table 2). The details of the two ALS subgroups, such as clinical diagnosis, clinical onset site, MFO, DPR and ALSFRS-r score, are shown in Table 2. Compared with the CSF IFN-γ levels (172.22±17.58 pg/ml) or serum IFN-γ levels (136.42±14.79 pg/ml) of the non-ALS patients respectively, both CSF IFN-γ levels (273.92±24.50 pg/ml and 385.21±16.34 pg/ml, respectively) or serum IFN-γ levels (262.82±30.91 pg/ml and 289.34±17.48 pg/ml, respectively) of ALS<12 m and ALS≥12 m subgroups were significantly increased (P<0.01). Moreover, a significant difference (p<0.01) was detected in the CSF IFN-γ levels between the ALS≥12 m (385.21±16.34 pg/ml) and ALS<12 m (273.92±24.50 pg/ml) subgroups. Furthermore, we found significant differences (p<0.01) in the CSF levels of IFN-γ (385.21±16.34 pg/ml) in the ALS≥12 m subgroup compared with the serum levels of IFN-γ either in the ALS≥12 m subgroup (289.34±17.48 pg/ml) or in the ALS<12 m subgroup (262.82±30.91 pg/ml). The CSF levels of IFN-γ in the ALS≥12 m subgroup were significantly elevated (p<0.01).

**Table 2:** Summary of samples from ALS patients (ALS) categorized according to MFO and from non-ALS patients (Non-ALS).

Sample information	ALS<12 m	ALS≥12 m	Non-ALS
**Cases (M/F)**	17 (12/5)	35(24/11)	31(19/12)
**Clinically definite/probable**	6/11	29/6	N/A
**Clinical onset, B/L**	5/12	9/26	N/A
**Age (years, mean±se)**	51.71±3.73	52.16±2.24	49.42±2.93
**MFO (months)**	7.41±0.72	24.89±2.33	N/A
**DPR**	1.36±0.39	1.43±0.19	N/A
**ALSFRS-r score**	37.18±0.97	35.77±1.11	N/A
**IFN-γ (pg/ml) (CSF)**	273.92±24.50*	385.21±16.34* # +	172.22±17.58
**IFN-γ (pg/ml) (Serum)**	262.82±30.91*	289.34±17.48*	136.42±14.79

ALS<12 m: the time from ALS onset to diagnosis was less than 12 months; AL≥12 m: the time from ALS onset to diagnosis was equal to or more than 12 months

* indicates a significant difference (p<0.01) in the IFN-γ levels between ALS and Non-ALS.

# indicates a significant difference (p<0.01) in the CSF IFN-γ levels between the ALS≥12 m and ALS<12 m subgroups.

+ indicates a significant difference (p<0.01) in the CSF IFN-γ levels in the ALS≥12 m subgroup compared with either the serum IFN-γ level in the ALS<12 m subgroup or the serum IFN-γ levels in the ALS≥12 m subgroup; mean±se.

**Fig 1:**
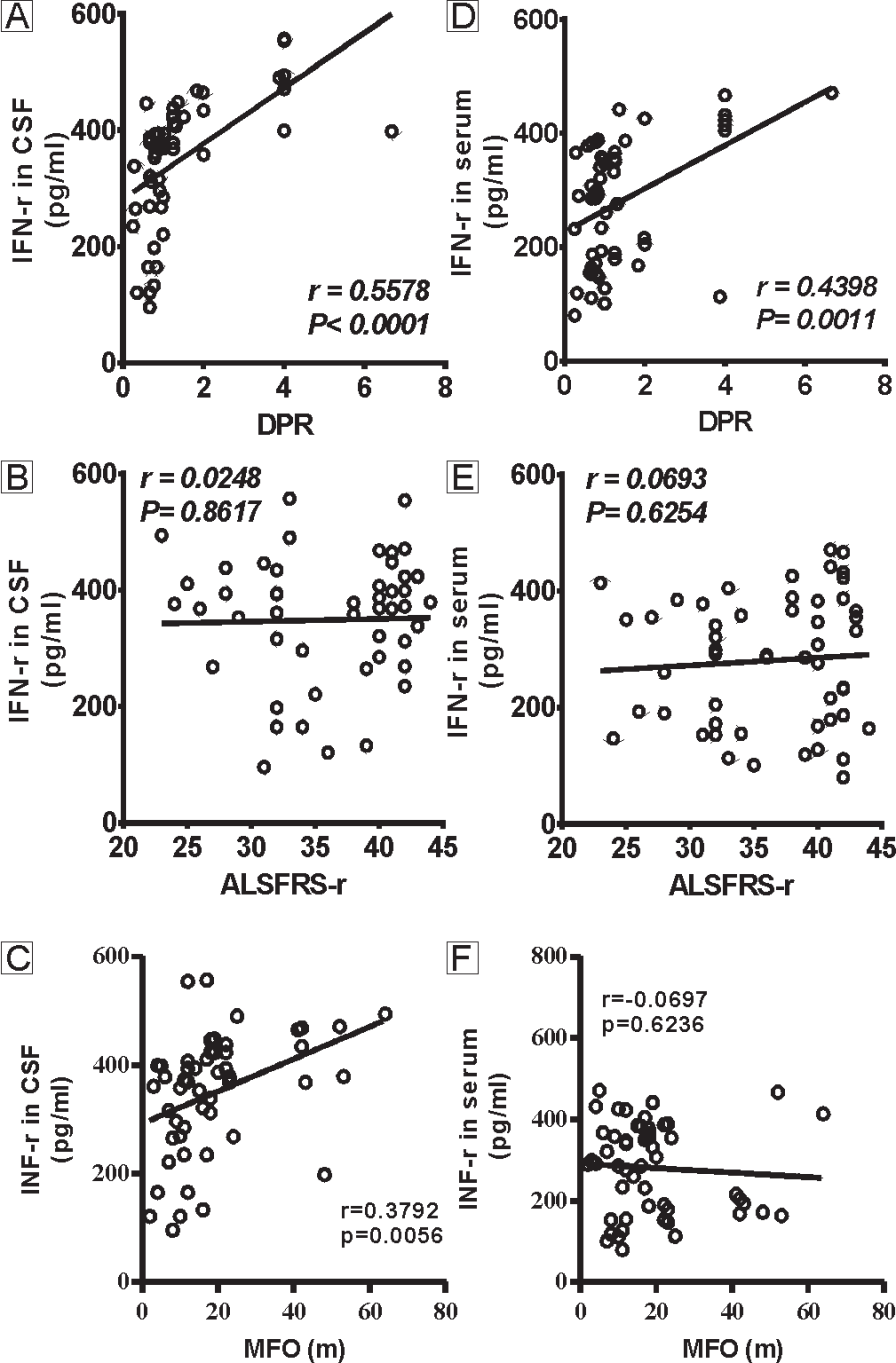
Correlations between the levels of IFN-γ and disease progression. Fig 1A-C show the correlations between the CSF IFN-γ levels and the DPR, ALSFRS-r, and MFO in the ALS patients. The IFN-γ levels in CSF positively correlate with the DPR (A) and the MFO (C) but not the ALSFRS-r score (B). Fig 1D-F show the correlations between the serum IFN-γ levels and the DPR, the ALSFRS-r score, and the MFO in the ALS patients. The IFN-γ level in serum significantly correlate with the DPR (D) but not the ALSFRS-r score (E) or the MFO (F).

The correlations of the CSF and serum IFN-γ levels with several parameters reflecting disease progression were analyzed in ALS patients (Fig 1). The IFN-γ levels in CSF positively correlated with the DPR (r = 0.5578, P<0.0001, Fig 1A) and MFO (r = 0.3792, P = 0.0056, Fig 1C) but not the ALSFRS-r score (r = 0.0248, P = 0.8617, Fig 1B). The IFN-γ levels in serum positively correlated with only the DPR (r = 0.4398, P = 0.0011, Fig 1D), not ALSFRS-r score (r = 0.0693, P = 0.6254, Fig 1E) or the MFO (r = -0.0697, P = 0.6236, Fig 1F).

**Fig 2:**
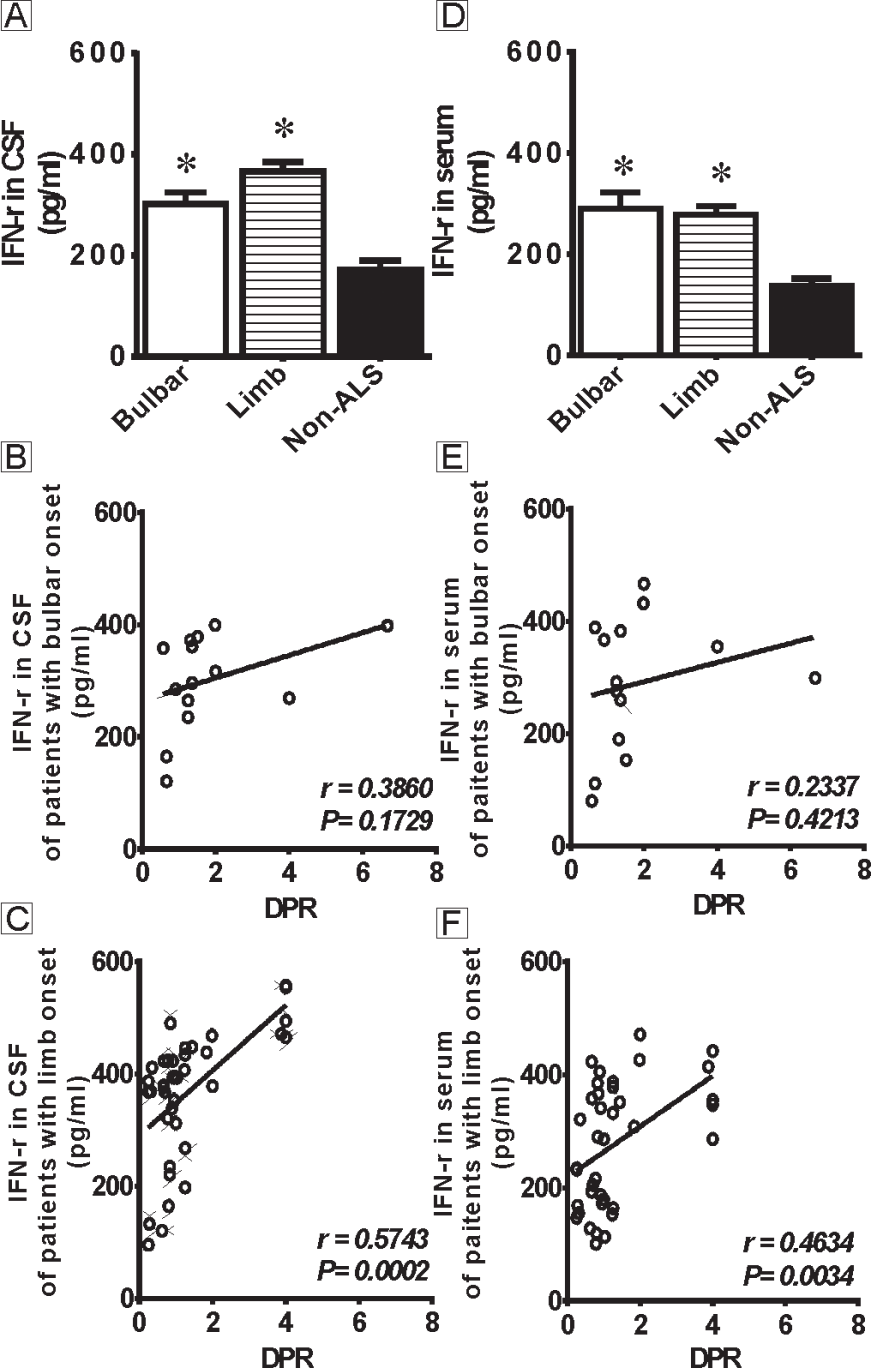
The levels of IFN-γ in CSF and serum of ALS patients with bulbar or limb onset and their correlations with disease progression. Fig 2A shows the levels of IFN-γ in CSF of ALS patients with bulbar or limb onset. There are significant differences in the CSF levels of IFN-γ in ALS patients (both bulbar and limb onset) compared with non-ALS patients (P<0.01), but no difference is observed between the bulbar and limb onset subgroups (P>0.05). Fig 2B-C show the correlations between the CSF IFN-γ levels and the DPR. The CSF IFN-γ levels do not correlate with the DPR in bulbar onset patients (B) but positively correlate with the DPR in limb onset patients (C). Fig 2D shows the levels of IFN-γ in serum of ALS patients with bulbar or limb onset. There are significant differences in the serum levels of IFN-γ in ALS patients (both bulbar and limb onset) compared with non-ALS patients (P<0.01), but no difference is observed between the bulbar and limb onset subgroups (P>0.05). Fig 2E-F show the correlations between the serum IFN-γ levels and the DPR. The serum IFN-γ levels do not correlate with the DPR in bulbar onset patients (E) but positively correlate with the DPR in limb onset patients (F). * indicates significant differences (p<0.01) in the CSF or serum IFN-γ levels between the ALS patients (both bulbar and limb onset) and the non-ALS patients.

Those findings indicated that the DPR may serve as a more important measure of disease progression in ALS patients and as a correlate of the IFN-γ levels. Thus, the second aim of this study was to further understand the correlations of the DPR with IFN-γ in ALS patients of bulbar or limb onset. All 52 ALS patients were re-categorized according to the site of onset. The CSF levels of IFN-γ are shown in Fig 2A. The CSF IFN-γ levels were markedly increased in the bulbar (301.29±22.84 pg/ml) and limb (366.32±18.55 pg/ml) onset subgroups compared with the non-ALS patients (172.22±17.58 pg/ml, P<0.01), but no difference was detected between the bulbar and limb onset subgroups (P>0.05). The DPR positively correlated with the CSF levels of IFN-γ in the limb onset subgroup (r = 0.5743, P = 0.0002, Fig 2C) but not in the bulbar onset subgroup (r = 0.3860, P = 0.1729, Fig 2B). Furthermore, as shown in Fig 2D, the serum IFN-γ levels were markedly increased in the bulbar (289.57±31.98 pg/ml) and limb (277.32±17.77 pg/ml) onset subgroups compared with the non-ALS patients (136.42±14.79 pg/ml, P<0.01), but no difference was observed between the bulbar and limb onset subgroups (P>0.05). The DPR correlated with the serum levels of IFN-γ in the limb onset subgroup (r = 0.4634, P = 0.0034, Fig 2F) but not in the bulbar onset subgroup (r = 0.2337, P = 0.4213, Fig 2E). This may due to the small numbers of ALS patients of bulbar onset.

**Fig 3:**
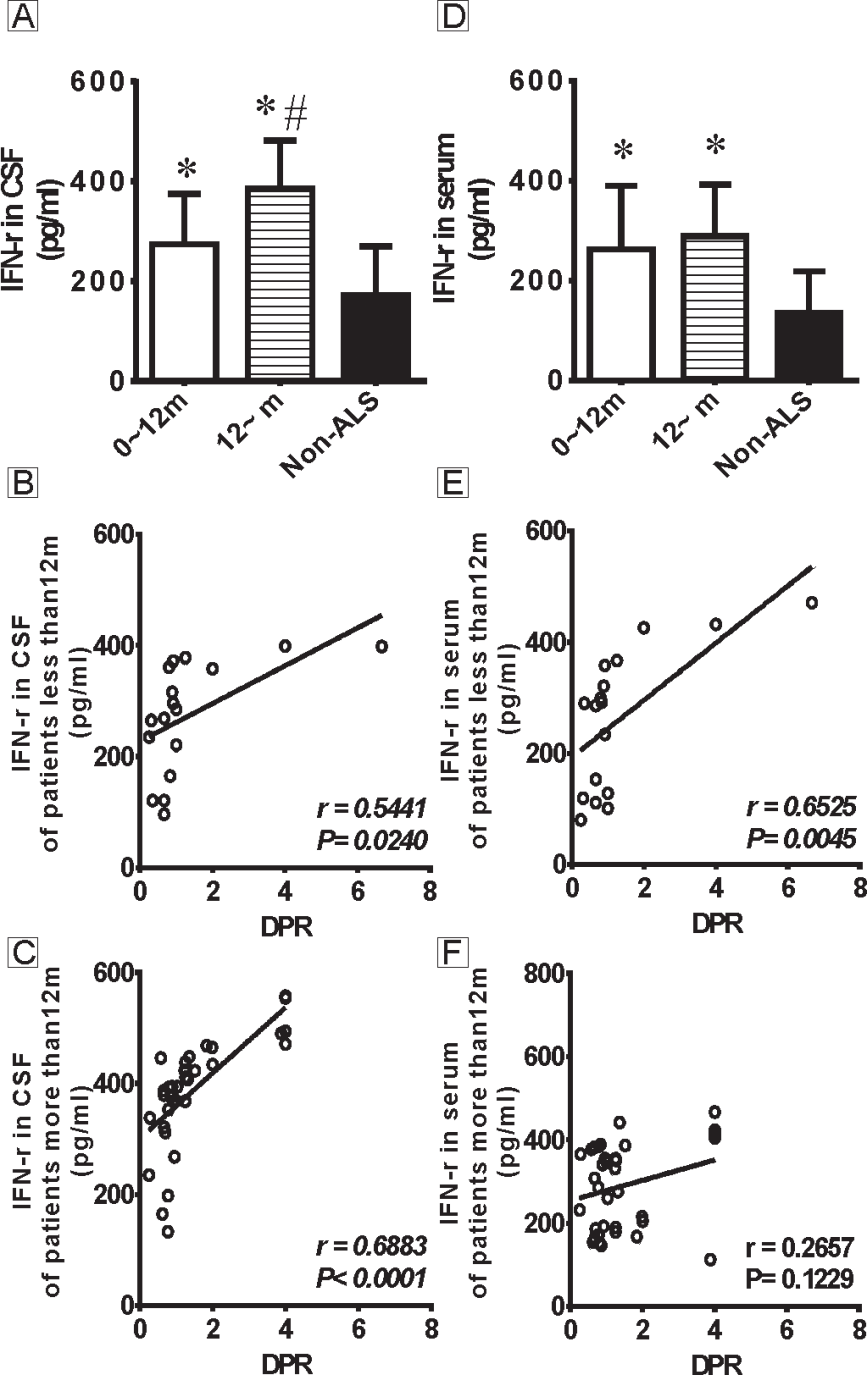
The levels of IFN-γ in CSF and serum of ALS patients stratified according to MFO and their correlations with disease progression. Fig 3A shows the levels of IFN-γ in CSF of ALS patients stratified according to MFO. There are significant differences in both ALS subgroups (ALS<12 m and ALS≥12 m) compared with the non-ALS patients (P<0.01), and a difference is detected between the ALS<12 m and ALS≥12 m subgroups (P<0.01). Fig 3B-C show the correlations between the CSF IFN-γ levels and the DPR in the ALS<12 m and ALS≥12 m subgroups. The CSF IFN-γ levels positively correlate with the DPR in both subgroups. Fig 3D shows the levels of IFN-γ in serum of ALS patients stratified according to MFO. There are significant differences in both ALS subgroups (ALS<12 m and ALS≥12 m) compared with the non-ALS patients (P<0.01), but no difference is observed between the ALS<12 m and ALS≥12 m subgroups (P>0.05). Fig 3E-F show the correlations between the serum IFN-γ levels and the DPR in ALS<12 m and ALS≥12 m subgroups. The serum IFN-γ levels positively correlate with the DPR in the ALS<12 m subgroup but not in the ALS≥12 m subgroup. * indicates significant differences (p<0.01) in the CSF or serum IFN-γ levels between the ALS patients (both ALS<12 m and ALS≥12 m) and the non-ALS patients. # indicates a significant difference (p<0.01) in the CSF IFN-γ levels between the ALS≥12 m and ALS<12 m subgroups.

The third aim of this study was to analyze the correlations of the DPR with the IFN-γ levels in CSF and serum of ALS patients with different MFOs. All 52 ALS patients were re-categorized according to the MFO. As shown in Fig 3A, the CSF IFN-γ levels was markedly increased in the ALS<12 m (273.92±24.50 pg/ml) and ALS≥12 m (385.21±16.34 pg/ml) subgroups compared with the non-ALS patients (172.22±17.61 pg/ml, P<0.01). Additionally, a significant difference in the CSF IFN-γ levels was detected between the two ALS patient subgroups (P<0.01). The DPR positively correlated with the CSF level of IFN-γ in both the ALS <12 m (r = 0.5441, P = 0.0240, Fig 3B) and ALS≥12 m (r = 0.6883, P<0.0001, Fig 3C) subgroups. As shown in Fig 3D, serum IFN-γ levels were markedly increased in both the ALS<12 m (262.82±30.91 pg/ml) and ALS≥12 m (289.34±17.48 pg/ml) subgroups compared with non-ALS patients (136.42±14.79 pg/ml, P<0.05), but no difference was observed between these two ALS patient subgroups (P>0.05). The DPR positively correlated with the serum IFN-γ level in the ALS<12 m subgroup (r = 0.6525, P = 0.0045, Fig 3E) but not in the ALS≥12 m subgroup (r = 0.2657, P = 0.1229, Fig 3F).

## Discussion

The present study demonstrated that the levels of IFN-γ were increased in both CSF and serum of ALS patients. Those findings were consistent with those of previous studies. Because the CSF and serum levels of IFN-γ correlated with disease progression, our findings supported the hypothesis that IFN-γ is involved in CNS inflammatory activity in ALS [8,10]. Accumulating evidence suggested a critical role of IFN-γ in ALS. Badu reported that the serum IFN-γ level was significantly elevated with disease progression in ALS patients from northern India [2]. Aebischer reported that the level of IFN-γ was increased in post-mortem tissues of the spinal cord from ALS patients in France [1]. Several studies reported the elevation of IFN-γ in CSF and serum of ALS patients compared to various control subjects [3,8,11]. In this study, we found not only the elevation of IFN-γ in CSF and serum in ALS patients compared to non-ALS patients but also a significant difference between the CSF and serum IFN-γ levels in ALS patients. Compared to the ALSFRS-r score and the MFO, the DPR is a more reliable measure associated with the change in the IFN-γ levels in both CSF and serum.

IFN-γ has been considered to act as a potent immunomodulatory cytokine released from natural killer (NK) cells, monocytes/macrophages, and T and B lymphocytes [12], NK cells are the primary source of IFN-γ in the periphery. In the CNS, IFN-γ is expressed by both astrocytes and motor neurons in patients and mice [13]. Astrocytes express IFN-γ-R in ALS patients [14]. Microglia and oligodendrocytes constitutively express IFN-γ-R at the mRNA and protein levels in culture but not *in vivo*, suggesting that the IFN-γ-astrocyte interaction plays a key role in the pathogenesis of ALS [14] [13]. Using human post-mortem tissues, marked upregulation of the IFN-γ receptor was found on activated astrocytes in ALS patients, suggesting that IFN-γ stimulates astrocytes to induce neurotoxicity [15]. IFN-γ activates astrocytes to release potentially neurotoxic products which are detrimental to neighbouring neurons [16]. IFN-γ acts by stimulating the death pathway of the LIGHT-LT-bR, triggering the LIGHT-independent death pathway or collaborating with other death-promoting factors [1,17]. IFN-γ can alter astrocytic metabolism by activating human microglial cells and can functioning in combination with IL-1b to activate human astrocytes, inducing the release of IL-12 by microglia via IL-6 [18–20]. The elevation of the IL-6 and IL-12 levels has been confirmed in ALS patients [21–24]. The above findings strongly suggest that IFN-γ is involved the pathology of ALS and that IFN-γ might contribute to the neuroinflammation and neurodegeneration underlying this disease.

Based on further investigation of the levels of IFN-γ in ALS of bulbar and limb onset, we found that the levels of IFN-γ in CSF and serum were elevated in both bulbar and limb onset ALS patients compared to non-ALS patients but that there was no difference between the bulbar and limb onset ALS patients. Moreover, the levels of IFN-γ in CSF and serum correlated with disease progression in the limb subgroup but not in the bulbar onset subgroup, suggesting that IFN-γ may serve as a reliable marker of early diagnosis and differentiation for ALS patients with limb onset but not bulbar onset. This result may reflect that other factors influence the mechanisms underlying ALS of bulbar and limb onset. It is also possible that distinct pathologic processes are related to the site of disease onset.

Little is known about the variation in the IFN-γ levels according to the different MFO and disease progress of ALS, especially in Chinese ALS patients.

Thus, we investigated the levels of IFN-γ in CSF and serum at two disease stages: less than and greater than 12 months from disease onset. We found that the IFN-γ levels were markedly elevated in CSF, but not in serum, at the later stage compared to the earlier stage. The CSF IFN-γ levels consistently correlated with disease progression, but the serum IFN-γ levels correlated with disease progression only at the earlier stage, not at the later stage. Those findings suggested that the elevation of the IFN-γ levels in serum at the earlier disease stage may result from the activation of peripheral immune cells such as T and B lymphocytes, natural killer cells, and monocytes/macrophages to release IFN-γ into the serum. In contrast, the levels of IFN-γ in CSF was markedly elevated at the later stage compared to the earlier stage. We suppose that the CSF IFN-γ levels could be affected by the function of the blood brain barrier. Moreover, it should be noted that in the CNS, IFN-γ is expressed by astrocytes and motor neurons at disease onset and during the symptomatic stage of disease; thus, it is reasonable to postulate that the IFN-γ levels in CSF at the earlier stage could be primarily attributable to IFN-γ release from these cells, which significantly increases at the later stage due to cell damage and disease progression. Collectively, these data suggested that IFN-γ might contribute to ALS pathogenesis and progression and that the CSF IFN-γ levels might serve as a more reliable marker of disease diagnosis and progression than the serum IFN-γ levels. On the other hand, the levels of IFN-γ were prominent at 24 months from ALS onset, reflecting a downstream event of systemic inflammation of this disease [2]. The levels of IFN-γ in serum were also reported in other different diseases, Medina *et al* reported that the IFN-γ level was higher in *P*. *vivax*-infected patients with increased disease severity [21], Liu *et al* found IFN-γ overcomes the resistance of tumor cell apoptosis by regulating the expression of apoptosis-related genes [22].

These results demonstrated that IFN-γ in CSF may serve as a biomarker of ALS differentiation and progression. CSF IFN-γ was a more reliable biomarker of disease diagnosis and progression than serum IFN-γ. However, the results are limited due to small numbers of ALS patients and limb onset was more commonly observed than bulbar onset. Further investigations could help to confirm the results from this preliminary report.
